# Variable fixation promotes callus formation: an experimental study on transverse tibial osteotomies stabilized with locking plates

**DOI:** 10.1186/s12891-020-03781-6

**Published:** 2020-12-03

**Authors:** Michael Plecko, Karina Klein, Katrin Planzer, Dirk Wähnert, Pascal Behm, Stephen J. Ferguson, Stefano Brianza, Vincent A. Stadelmann, Brigitte von Rechenberg

**Affiliations:** 1 Trauma Hospital Graz (UKH), Goestinger Strasse 24, 8021 Graz, Austria; 2 Musculoskeletal Research Unit (MSRU), Vetsuisse Faculty, University of Zurich, Zurich, Switzerland; 3 Protestant Hospital of Bethel Foundation, Department of Trauma and Orthopedic Surgery, Bielefeld, Germany; 4 Institute for Biomechanics, ETH, Zurich, Switzerland; 5 Biomech Innovations AG, Aarbergstrasse 5, CH-2560 Nidau, Switzerland; 6 Schulthess Clinic, Department of Research and Development, Zürich, Switzerland; 7 Center for Applied Biotechnology and Molecular Medicine (CABMM), Vetsuisse Faculty, University of Zurich, Zurich, Switzerland

**Keywords:** Variable fixation, VFLS, Variable fixation locking screw, Fracture healing, Fracture dynamization, Callus formation, Fracture complications, Non-unions, Delayed unions

## Abstract

**Background:**

A new locking screw technology, named variable fixation, has been developed aiming at promoting bone callus formation providing initial rigid fixation followed by progressive fracture gap dynamisation. In this study, we compared bone callus formation in osteotomies stabilized with standard locking fixation against that of osteotomies stabilized with variable fixation in an established tibia ovine model.

**Methods:**

A 3 mm tibial transverse osteotomy gap was stabilized in three groups of six female sheep each with a locking plate and either 1) standard fixation in both segments (group LS) or 2) variable fixation in the proximal and standard fixation in the distal bone segment (group VFLS_3_) or 3) variable fixation in both segments (group VFLS_6_). The implantation site and fracture healing were compared between groups by means of radiologic, micro tomographic, biomechanical, and histological investigations.

**Results:**

Compared to LS callus, VFLS_3_ callus was 40% larger and about 3% denser, while VFLS_6_ callus was 93% larger and its density about 7.2% lower. VFLS_3_ showed 65% and VFLS_6_ 163% larger amount of callus at the cis-cortex. There wasn’t a significant difference in the amount of callus at the cis and trans-cortex in groups featuring variable fixation only. Investigated biomechanical variables were not significantly different among groups and histology showed comparable good healing in all groups. Tissues adjacent to the implants did not show any alteration of the normal structure in all groups.

**Conclusions:**

Variable fixation promoted the formation of a larger amount of bone callus, equally distributed at the cis and trans cortices. The histological and biomechanical properties of the variable fixation callus were equivalent to those of the standard fixation callus. The magnitude of variable fixation had a biological effect on the formation of bone callus. At the implantation site, the usage of variable fixation did not raise additional concerns with respect to standard fixation. The formation of a larger amount of mature callus suggests that fractures treated with variable fixation might have a higher probability to bridge the fracture gap. The conditions where its usage can be most beneficial for patients needs to be clinically defined.

## Background

Projection on population aging suggests that the cost and the societal burden of complicated fracture cases will not be sustainable in the coming years [1–5]. This must stimulate the usage of surgical techniques and the development of devices fostering fracture healing at the first surgery as the most effective strategy to prevent complications. The preservation of the blood supply through minimally invasive surgical access and functional reduction according to biological fracture healing principles are key factors for treatment success through secondary fracture healing. Implant choice, construct and loading determine fracture stability and play a key role in callus formation. However, increasing evidence suggests that different phases of fracture healing may benefit from different levels of stability, respectively micromotion. In particular, the inflammatory phase seems to profit from a stable mechanical environment [6–9] fostering blood vessels formation [10–13] and the differentiation of mesenchymal cells towards an osteogenic lineage rather than a chondrogenic lineage [14, 15]. In turn, mechanical stimulation during callus formation and remodeling may enhance stabilization through stimulating vascular network remodeling [13] and the formation of additional cartilage and bone [9].

**Fig. 1:**
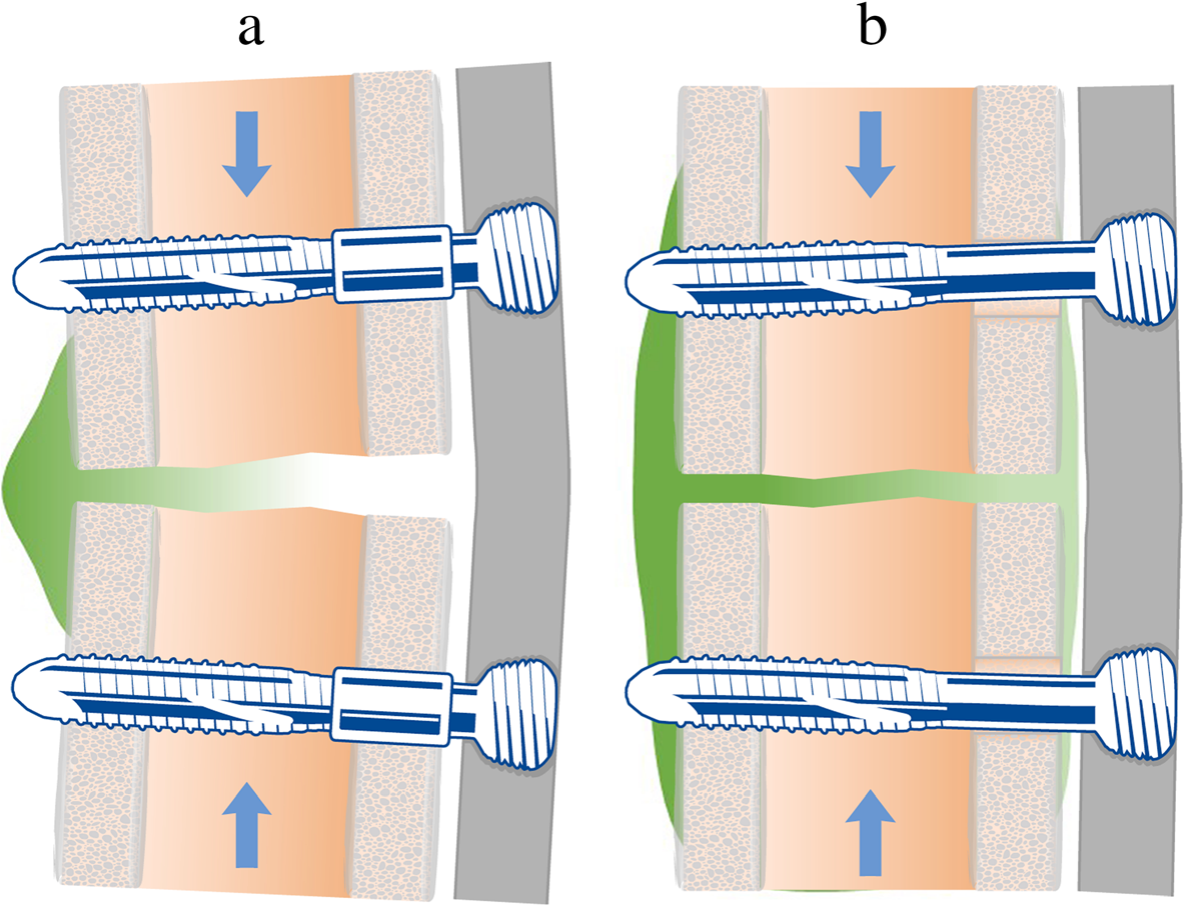
schematic representation of longitudinal sections of bone segments implanted with variable fixation. In grey the bone plate, in green the forming bone callus and in blue the axial loading on the construct. Panel **a** - At the beginning of the treatment, in the inflammatory phase, the rigidity of the fixation and the displacements at cis and trans cortices are equivalent to those provided by standard locking screws [16]. These conditions are known to promote the formation of bone callus mainly at the trans-cortex. Panel **b** - VFLS sleeve degrades through superficial hydrolysis following the profile characteristic of resorbable polymers of the same family [17–20], namely progressive and spaced over time molecular weight loss, loss of mechanical properties and loss of mass. As the sleeve starts losing mechanical properties in the callus formation phase, the entire fixation progressively becomes more flexible. The displacements at cis and trans cortices progressively change, with a marked increase of interfragmentary displacements at the cis-cortex [16]. These changes aim at progressively straining the entire fracture gap in the “window of opportunity”, namely a strain range promoting the formation of bone callus

Aiming at combining these requirements, an incremental innovation of the standard locking technology, named variable fixation, has been developed allowing for stepwise and predictable modulation of construct stiffness and interfragmentary movements [16]. Variable Fixation Locking Screw (VFLS) was designed to provide rigid fixation during the inflammatory phase and progressively dynamize the forming callus by means of a poly-(lactide-co-glycolide) sleeve positioned in the cis cortex. The degradation of the sleeve changes the relative stability of bone fragments giving progressively way to micromovement during the phase of callus formation (Fig. 1).

The goal of this study was to answer the following questions about variable fixation technology: 1) Is variable fixation promoting the formation of a larger amount of bone callus and how is it affecting its distribution around the bone? 2) Is there a difference when using variable fixation on one bone segment only or on both segments? 3) Is the maturation of this callus comparable to that of standard locking fixation? 4) Is variable fixation raising concerns at the implantation site? For this purpose, we compared bone callus formation in standardized osteotomies stabilized with standard fixation against that of osteotomies stabilized with variable fixation and against that of osteotomies stabilized with a combination of the two fixation methods.

## Methods

### Surgery

**Fig. 2:**
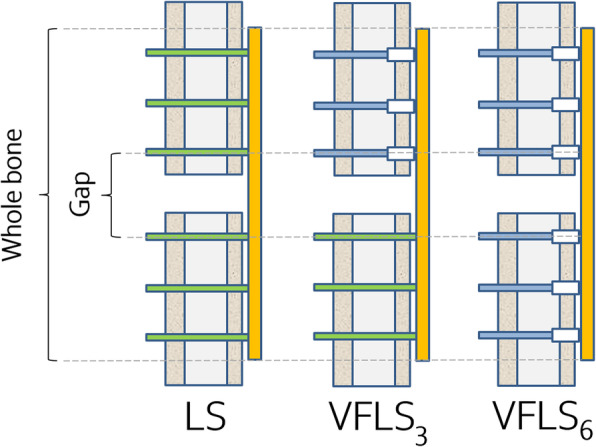
Schematic representation of the three groups. In brownish colors longitudinal cross-sections of the proximal and distal tibia segments; in yellow the bone plate; in green the standard locking screws, and in blue the Variable Fixation Locking Screws featuring in white their resorbable sleeve. On the left, the regions of interest assessed by micro CT are visually defined

Twenty adult Swiss-Alpine female sheep with the average of 2.7 years age and 72.9 ± 6.0 kg mass were used in this study. Animals were acquired from a private source (Knüsel, Küttigen, Switzerland). All experiments were conducted according to Swiss laws for animal welfare (TSchG 455) and granted by the local veterinary authorities (license # ZH 071/17). Animals and treated limbs were randomly selected and evenly allocated to three treatment groups with six sheep each after 12.4 days of acclimatization on average. Surgeries were alternated on right and left tibiae. A 4.5/5.0 broad six-hole, locking compression plate (426.561, Synthes, Oberdorf, Switzerland) was implanted with three different combinations of screws (Fig. 2). In group VFLS_3_, three Variable Fixation Locking Screws (S540032S, 5.0 mm Variable fixation Locking Screw, Ti alloy, Biomech Innovations, Nidau, Switzerland) were implanted in the proximal and three standard locking screws (413.332, 5.0 mm locking screw, Ti alloy, DePuy Synthes, Oberdorf, Switzerland) were implanted in the distal segment. Both the proximal and distal segments were implanted with three locking screws in group LS and with three Variable Fixation Locking Screws in group VFLS_6_.

Variable fixation has been characterized in a previous biomechanical investigation on constructs featuring combinations of technologies similar to those tested here in vivo [16]. In vitro, we have shown that, at the beginning of the treatment, the stability offered by VFLS_3_ and VLFS_6_ is equivalent to that of standard LS technology. In VFLS groups, sleeve resorption led to a significant decrease in construct stiffness and to significantly larger interfragmentary displacements, with a remarkable increase at the cis cortex. All these changes nicely commensurate to the combination of technologies, with VFLS_3_ providing intermediate values between LS and VFLS_6_.

**Fig. 3:**
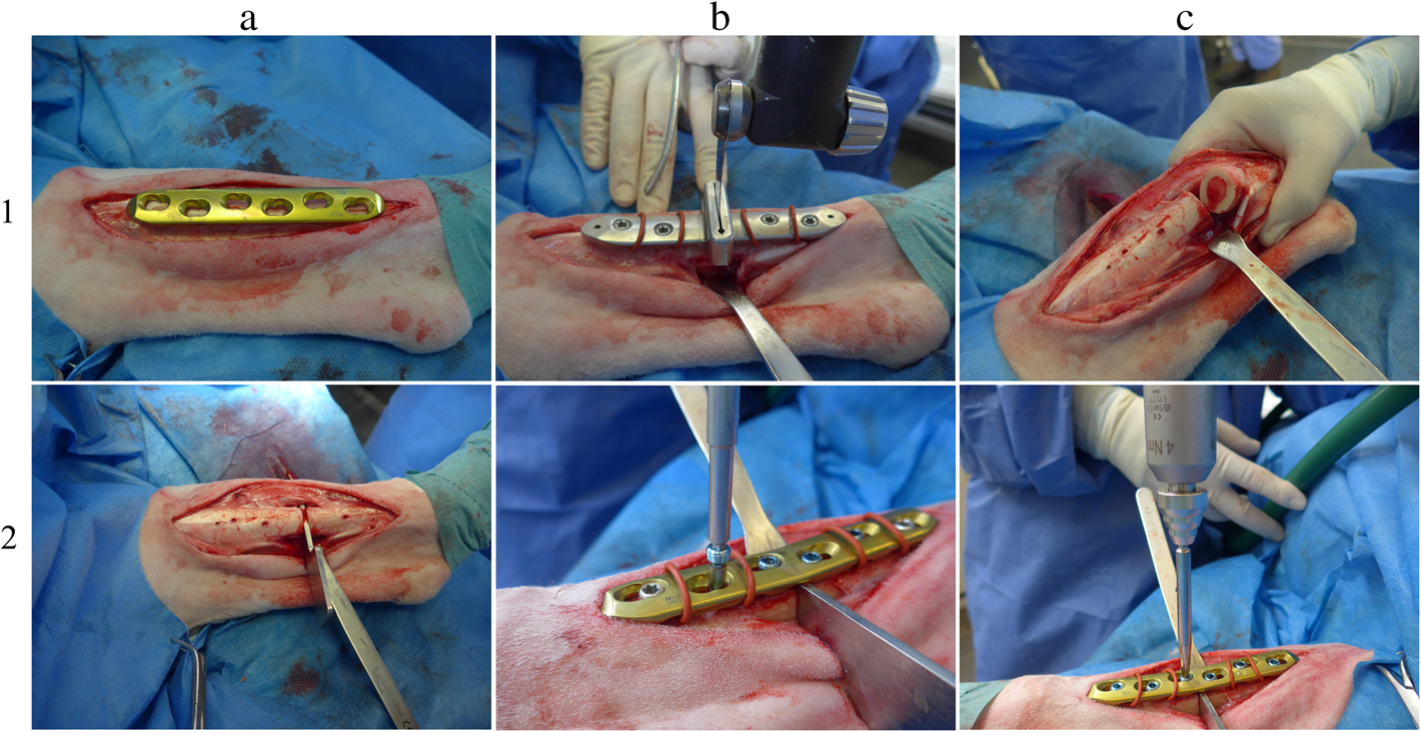
The broad 6-hole LCP was slightly contoured to fit the tibial shaft (panel **a**1) and a custom cutting guide was temporarily fixed to the intact tibia. An osteotomy was performed through the guiding slots (panel **b**1-**c**1). The fragments were then repositioned and fixed with the six-hole plate and either combination of screws with the 3-mm distance metallic holder in place (panel **a**2-**c**2)

Surgery and all associated procedures strictly followed an established protocol [21] that allowed consistently fixing the tibial segments with a 3 mm parallel gap in all animals. Briefly, a 15 cm skin incision was performed on the medial aspect of the tibia and the broad 6-hole locking compression plate was slightly contoured to fit the tibial shaft. A custom cutting guide, was temporarily fixed to the intact tibia with four mono-cortical 4 mm screws (L16-18 mm, steel, DePuy Synthes 02.204.016–18). Four rubber rings allowed to position the plate at a standard distance from the periosteum. An oscillating saw (Synthes, saw blade 519.150, 70/49*14*0.6/0.4 mm) was used to perform the osteotomy through the guiding slots under constant irrigation with 0.9% saline solution. After removal of the custom cutting guide, the fragments were repositioned and fixed with the six-hole plate and either combination of screws with the 3-mm distance holder in place to further ensure a standardized parallel gap. All devices have been implanted using the instruments and torque recommended by the manufacturers and soft tissues were closed with resorbable suture material (Fig. 3). After surgery a full cast was applied protecting the fixation while allowing full weight bearing directly after surgery. Sheep were kept in small pens and a standard suspension system for 3 weeks. Starting at week three cast changes were performed in combination with weekly standardized radiographs taken in three projections (anteroposterior and mediolateral ±5°) until sacrifice. Finally, fluorescence dyes were administered subcutaneously to assess new bone deposition and remodeling during the healing period. Calcein green (5 mg/kg BW) was injected at 3 weeks, xylenol orange (90 mg/kg BW) at 6 weeks and oxytetracycline, (20 mg/kg BW) 48-72 h prior to sacrifice.

At 9 weeks all sheep were slaughtered in the hospital’s officially accredited slaughter by a professional butcher facility according to the Swiss law (VSFK, SR 817.190 and VHyS, SR 817.190.1). They were slaughtered using a cartridge driven captive bolt gun to achieve unconsciousness. Immediately thereafter, they were completely bled by cutting the main blood vessels in the neck area. All treated limbs were dissected documenting the macroscopic appearance of soft and hard tissues around implants. Local draining poplitei and inguinal lymph nodes of treated and contralateral limbs were first macroscopically examined for changes in size, color, consistency and, after harvesting, sent for histological assessment. Macroscopic assessment of the implants included overall stability of the fixation, locking of each individual screw, callus formation also over the implants, fibrosis and specifically metallosis around the implants. Finally, implants were removed and the operated and contralateral tibiae were sent for further investigations wrapped in hydrated gauzes.

### Radiologic evaluation

Weekly radiographs (week 3 to 9) were semi-quantitatively scored by two independent reviewers for cortical callus formation at the cis- and trans-cortex. There, callus formation at the periosteal and endosteal site were assessed focusing on bridging of the 3 mm gap. In the slightly oblique views, the cranial and caudal callus formation was scored separately. Additionally, opacity of the callus was recorded as well as the different reaction of the cortical bone around the tip of the screws as image of local cortical bone activation due to possible micromotion. Last, the Rust score was assessed for each osteotomy. Quantitative analysis of all radiographs (week 3–9) was performed using the imaging software (OsiriX) measuring the total callus area (cm^2^).

### Micro computed tomography

Bone samples wrapped with hydrated gauze were scanned with a cone-beam microCT (XtremeCT II, SCANCO Medical AG, Brüttisellen, Switzerland) with the x-ray tube operated at 68 kVp, 1470 μA; 900 projections/180° were acquired with 43 ms shutter time. The slices were reconstructed across an image matrix size of 1654 × 1654 voxels, with a nominal voxel size of 60.7 μm. A Gaussian filter was used to minimize noise and a thresholding algorithm was used for segmentation of bone (>1000mgHA/ccm) relative to background and for segmentation of callus (250-1000mgHA/ccm) relative to adjacent native bone. Bone and callus masks were refined using a custom series of opening and closing transformations. The image processing algorithms were developed with EasyIPL, a high-level library of macros using the scanner software (SCANCO Image Processing Language, IPL and HP OpenVMS Digital Command Language, DCL). Parameters of interest were bone and callus volume and density, the profile of the cross-sectional polar moment of inertia (pMOI) along the bone major axis, the callus volume at the cis and trans-cortex. Parameters were calculated in two regions of interest: the whole bone, defined as the sample volume under the plate, and the gap, defined as the sample volume between the proximal and distal screw adjacent to the osteotomy gap (Fig. 2).

### Biomechanical testing

Non-destructive torsion tests were performed. Operated and contralateral tibiae were tested in torsion on an Instron® E10000 testing machine. Axial load and torque were measured with a calibrated Instron® Load Cell ±10 kN / 100 Nm and recorded through the Instron® Console 8.4 software. Each tibia was fixed to the test frame using standard polymethylmethacrylate embedding and constantly kept moist using soaked tissues. Samples were tested in angular displacement control at 5 degree/min and under 20 N axial load. The machine stopped as a 3 Nm drop in recorded torque was detected in order to allow further histological investigations. Data were recorded at 20 Hz. Torque (Nm) and angular displacement (degree) at failure, apparent stiffness (Nm/degree) in the linear region of the loading curve, yield point (Nm) and energy to failure (Nm*degree) were determined. Variables of interest for each operated tibia were recorded as absolute values as well as normalized to the values measured for the contralateral limb.

After testing, all samples were cut in smaller pieces including the osteotomy site and the first proximal and distal screw holes and were brought to the histology laboratory fixed in 50% ethanol.

### Histology

Four investigations were performed: 1) analysis of the draining lymph nodes, 2) biocompatibility analysis of the local tissue effects according to ISO10993-6:2016, 3) histomorphometric measurements of callus formation in ground sections and 4) evaluation of bone deposition and remodeling using the fluorescent sections.

The inguinal and popliteal lymph nodes of treated and non-treated limbs of the VFLS_6_ and LS group were fixed in 4% formalin, routinely embedded in paraffin and stained with H&E for qualitative evaluation of structural changes and non-local cell content. Particular attention was paid to inflammatory cells and the presence of foreign material from the polymer sleeve and/or local metallosis. The Ziehl-Neelsen staining was used to determine the presence of acidophilic bacteria. Lymph nodes of VFLS_3_ were not evaluated due to the combined usage of different screws in the same animal.

After fixation of the non-decalcified bone samples in 50% ethanol, they were further fixed in an ascending series of ethanol under light protection. Thereafter, samples were defatted in xylene and subsequently infiltrated in liquid polymethylmetacrylate (PMMA) until blocks were hardened. The polymerized samples were then cut lengthwise in the axis and midline of the screws using an Exact 310 saw (EXAKT Technologies, Inc. Oklahoma City, US). Ground sections were polished and then surface stained with toluidine blue, thin sections (5 μm) were cut with a microtome (Leica RM 2155, Leica Instruments GmbH, Nussloch, Germany) and after mounting in glass slides stained with toluidine blue or von Kossa/McNeill. Toluidine blue staining allows to distinguish old from newly built bone due to its color intensity.

The evaluations of local biocompatibility (inflammation and tissue response) were performed on hematoxylin-eosin, toluidine blue and von Kossa stained thin sections (*N* = 138) using a light microscope (microscope Leica DMR system). The evaluation was performed by two independent observers. Assessment of biocompatibility parameters of VFLS screws in comparison to the standard locking screw was evaluated in the area of the cis- and trans-cortex screw holes including bone marrow cavity. The evaluation was performed comparing VFLS_6_ group screws to LS group screws as well as the VFLS screws and locking screws implanted in the same animals (VFLS_3_ group).

Software based quantitative histomorphometry was conducted on digital images of interactively color-highlighted toluidine blue stained ground sections captured with a microscope (Leica Z6 APOA, Leica DFC 420C, Glattbrugg, Switzerland). Measurements quantified the percentage of old and new bone, and non-bone (non-bone containing tissue like fibrous tissue, fat, bone marrow tissue) on the total section (between the proximal and distal screw adjacent to the osteotomy gap) and, in the gap, on the cis-, on the trans-cortex and on the endosteal area.

Fluorescent sections of the gap area were recorded and quantitatively evaluated with an image software (Fiji, ImageJ) for differences of dye integration between groups and at different time points (calcein green at 3 weeks, xylenol orange at 6 weeks and oxytetracycline at 9 weeks post-surgery).

### Statistical analysis

Sample size has been chosen on a power analysis of previous data derived from similar experimental measurements with this same model [21]. The a priori exclusion criteria were: implants not loaded due to incorrect positioning, loss of screw to plate rigid connection or not weight bearing animals. Statistical analysis was performed using parametric or non-parametric test methods depending on the normal distribution (ANOVA or Signed rank test) and appropriate post-hoc testing (in e.g. Tukey). All statistical analyses were performed using the software SPSS (Version 2.5) or R.

## Results

All surgeries went according to study design. Initially, in animals featuring a very small medullary cavity, inserting VFLS too close to the anterior or posterior endosteal wall caused a damage to the sleeve. In these cases, damaged screws were replaced by new screws. With an optimization of the screw cutting edges screws could be easily inserted. Healing of osteotomies were complete in all sheep. Two sheep of the VFLS_6_ group were replaced by the two reserve animals (*n* = 6 for all three groups). Reasons for exclusion were: 1) a screw implanted into the fracture gap and 2) one sheep constantly lying on the ground despite it could walk without signs of discomfort. This sheep developed a cutaneous lesion around the sternal area and was excluded because the loading on the operated limb was not comparable to the other sheep. Implant failure or loosening was not observed during the in-life phase and no problems were recorded during implants removal.

### Macroscopic evaluation at sacrifice

The tissue adjacent to the implants did not show alteration of the normal structure in all three groups. No hematoma, edema, encapsulation, and/or other additional gross findings were recorded. No remnants of the resorbable sleeve were detected. All screws were locked except the screw placed into the osteotomy gap. Metallosis was detected around 56% of the screws. There was a slight but not statistically significant tendency for more pronounced metallosis in the reference group LS. The 3 mm gap was bridged with abundant callus in all animals. No macroscopic abnormalities were found in the harvested lymph nodes.

### Radiologic evaluation

**Fig. 4:**
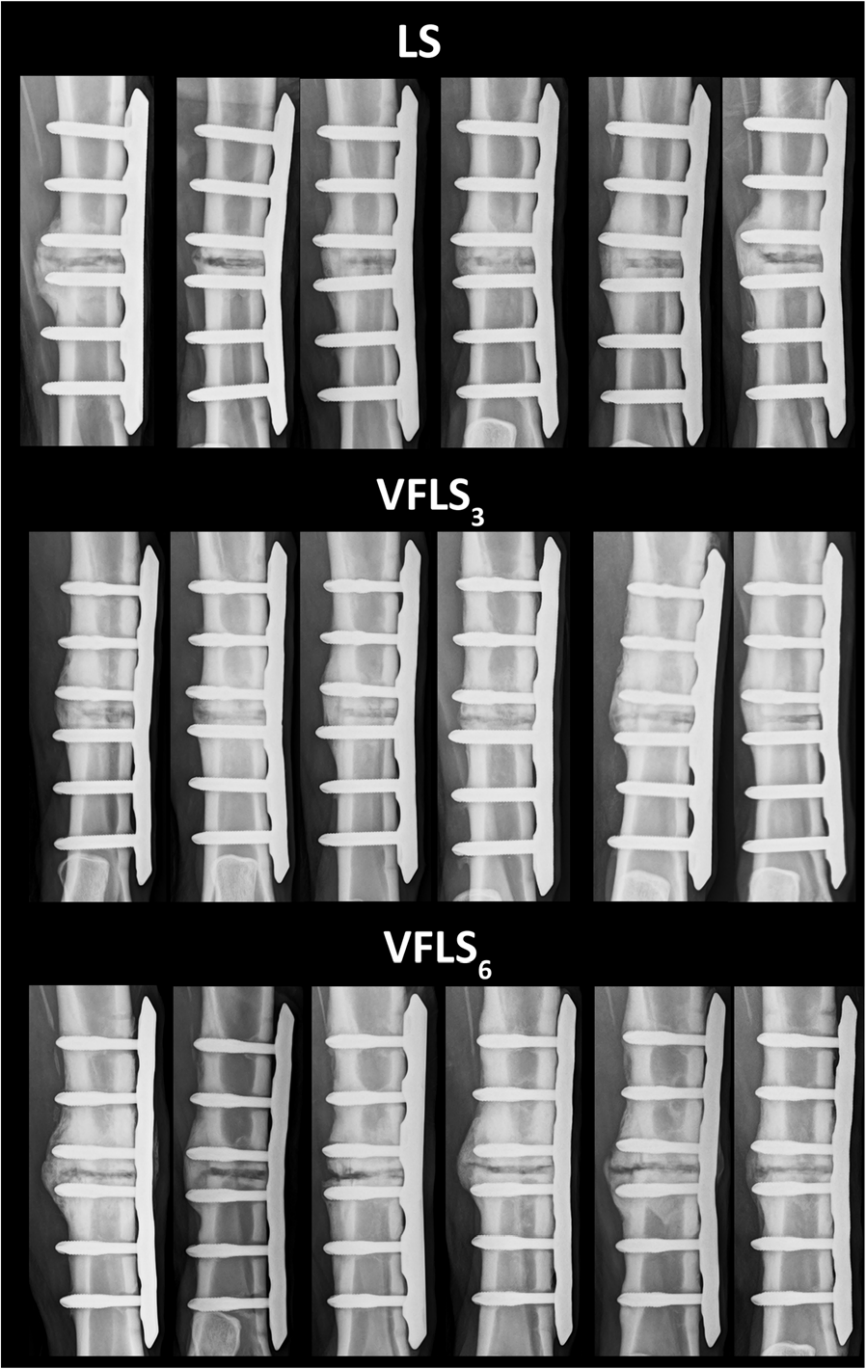
anterior posterior projections at 9 weeks for all sample included in the study. Notice the evident difference in activation of the cis and trans-cortex among groups and bone segments. In bone segments implanted with VFLS the trans-cortex is thickened and shows radiological signs of activation both at the medullary and periosteal sides. The superimposition of abundant callus creates a slightly different aspect of the cortices and different cortices/forming callus grays intensity ratio with respect to the radiologic images we’re used to see using standard locking screw. The establishment of new standard assessment criteria might be necessary

Radiologic analysis proved valuable to document for the first time the appearance of bones treated with variable fixation (Fig. 4). It allowed semi-quantitative evaluation assessing overall bone reaction, callus formation and bone resorption close to the implants. Overall, the VFLS_6_ showed lower semiquantitative scores compared to the VFLS_3_ and LS groups. The VFLS_3_ showed a similar score pattern than the LS group. Significant differences, however, were found mainly at week 6 and 9 between VFLS_3_ and LS compared to VFLS_6_ and only in the callus area of the trans-cortex. Bone activation of VFLS_6_ group was significantly larger than of LS groups until week 7 (all *p* ≤ 0.04).

Quantitative evaluation of callus formation proved difficult and showed very low accuracy and repeatability. The callus area had to be pinpointed manually by following its outline with a design tool giving way for inaccuracies. Therefore, results have to be interpreted with great caution. Basically, results showed higher mean callus values for VFLS_6_ in the anteroposterior and anterolateral projection and higher values of the VFLS_3_ group throughout all time points in the posterolateral projection. Due to high standard deviations, no significant differences were found for all time points.

#### Micro computed tomography

**Fig. 5:**
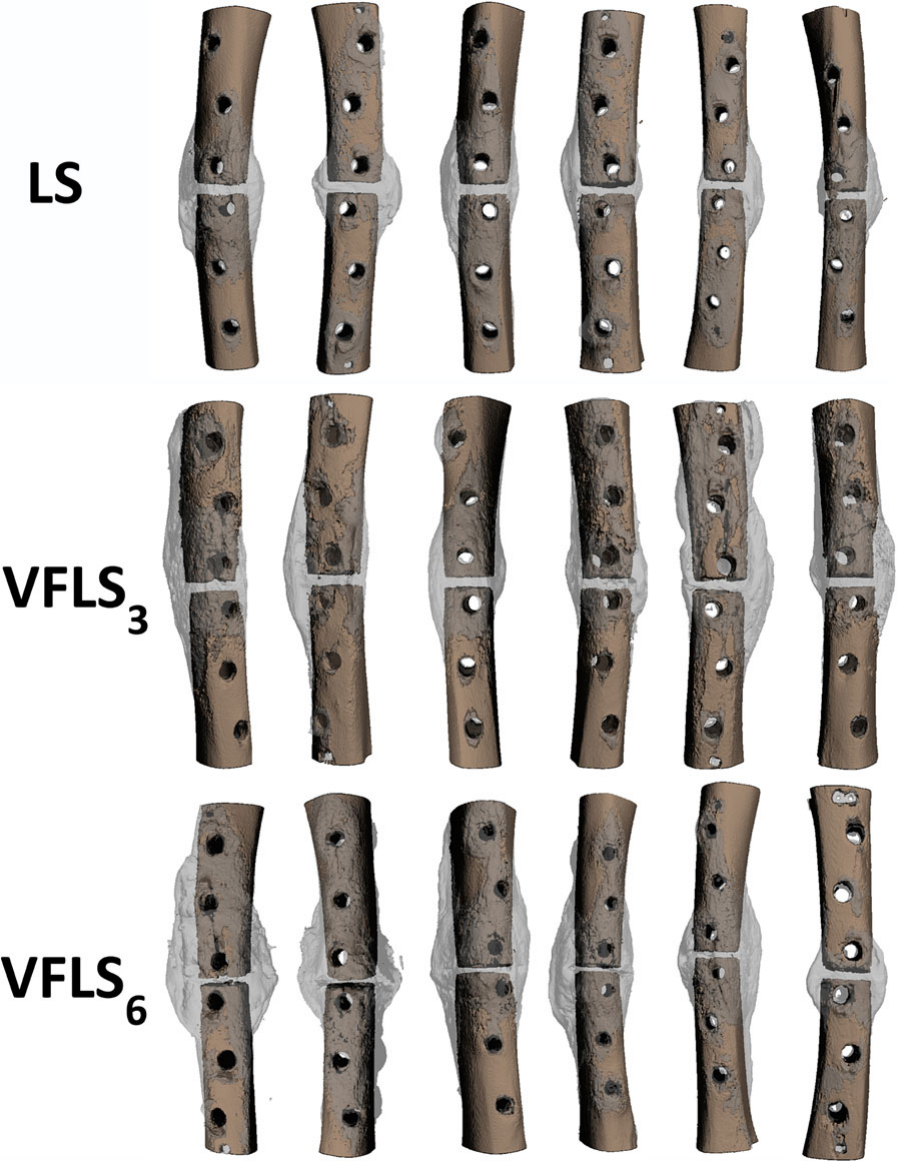
3D reconstruction of all tibiae included in the study visually showing the different amount and distribution of newly formed bone callus (in gray)

The global morphometric analysis using the subjective segmentation workflow was performed successfully and accurately depicted callus versus cortical bone. It allowed 3D-rendered reconstructions highlighting the callus area and cortical bone area for all operated tibia samples. Furthermore, the mature callus volume, representing the callus portion that was highly mineralized, could be successfully determined. This was a particularly difficult region to select as standard semi-automated density thresholding segmentation would have considered it equivalent to cortical bone, therefore a slice by slice evaluation was used (Fig. 5). The overall volume and bone density of the native tibiae were not significantly different among groups.

##### Whole bone -

**Fig. 6:**
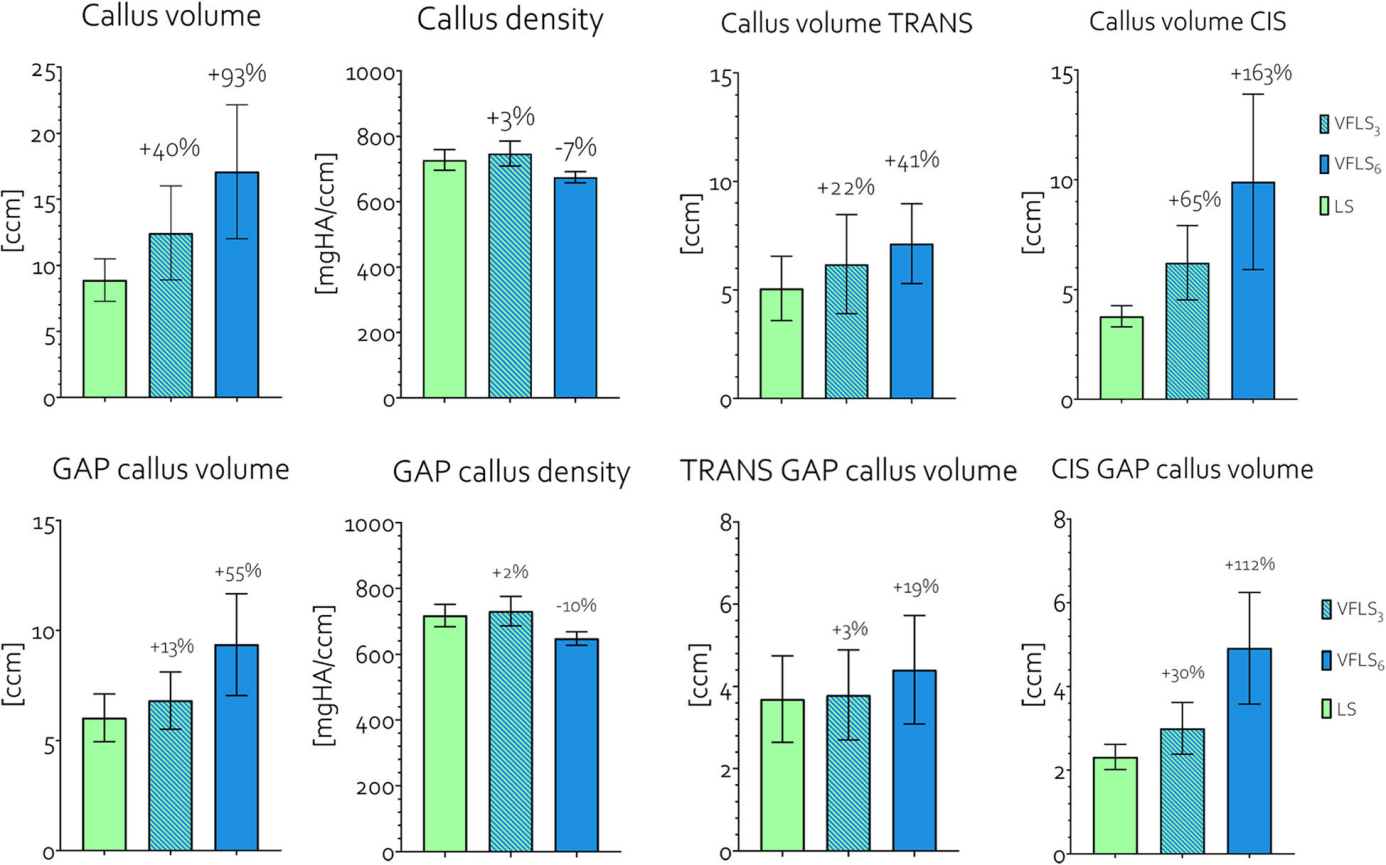
Comparison of bone callus formation between groups LS, VFLS_3_ and VFLS_6_ at the whole bone and at the gap level. The percentages are calculated with respect to the values recorded in group LS

VFLS_3_ callus was 40% larger in volume and featured 3% higher density while VFLS_6_ callus was 93% larger in volume and featured 7% lower density with respect to LS callus. While there was a similar amount of callus at the trans-cortex among groups, at the cis-cortex VFLS_3_ callus volume was 65% larger and that of VFLS_6_ was 163% larger than LS callus (Fig. 6, Table 1). On the whole bone, there was no significant difference between the callus at the cis and trans-cortex within each group.

**Table 1:** mean and SD (approximated values) of absolute data measured on at the whole bone and at the gap level for the three groups. Anova and post-hoc test show where there was a significant difference.

**Whole bone**	**LS**	**VFLS**_**3**_	**VFLS**_**6**_	**Anova**	**Post-hoc**
Callus volume [ccm]	8.9 ± 1.6	12.5 ± 3.5	17.1 ± 5.1	*p* = 0.0056	VFLS_6_/LS (*p* = 0.0042)
Callus density [mgHA/ccm]	728 ± 32	748 ± 38	675 ± 17	*p* = 0.0025	VFLS_6_/LS (*p* = 0.022) VFLS_6_/VFLS_3_ (*p* = 0.0024)
Trans callus volume [ccm]	5.1 ± 1.5	6.2 ± 2.3	7.1 ± 1.8	*p* = 0.200	n.a.
Cis callus volume [ccm]	3.8 ± 0.5	6.2 ± 1.7	9.9 ± 4.0	*p* = 0.0027	VFLS_6_/LS (*p* = 0.0020)
**GAP**	**LS**	**VFLS**_**3**_	**VFLS**_**6**_	**Anova**	**Post-hoc**
Callus volume [ccm]	6.0 ± 1.1	6.8 ± 1.3	9.3 ± 2.3	*p* = 0.0087	VFLS_6_/LS (*p* = 0.0089) VFLS_6_/VFLS_3_ (*p* = 0.0045)
Callus density [mgHA/ccm]	717 ± 34	730.8 ± 44.9	647.1 ± 20.5	*p* = 0.0016	VFLS_6_/LS (*p* = 0.0083) VFLS_6_/VFLS_3_ (*p* = 0.0021)
Trans callus volume [ccm]	3.7 ± 1.0	3.8 ± 1.1	4.4 ± 1.3	*p* = 0.529	n.a.
Cis callus volume [ccm]	2.3 ± 0.3	3.0 ± 0.6	4.9 ± 1.3	*p* = 0.0003	VFLS_6_/LS (p = 0.0003) VFLS_6_/VFLS_3_ (*p* = 0.0044)

##### Gap -

**Fig. 7:**
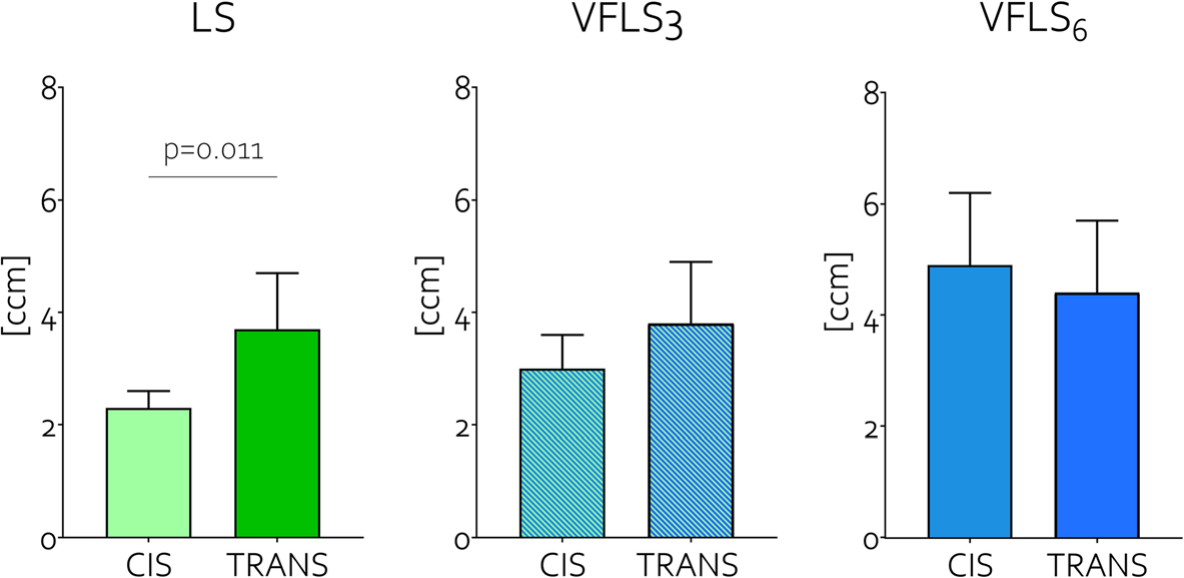
Amount of callus detected at the trans and cis-cortex in samples treated either with rigid fixation (LS) or different magnitudes of variable fixation (VFLS_3_ and VFLS_6_) in a 3 mm gap. At 9 weeks, a significant difference between cis and trans-cortex callus was found in the LS group but not in the VFLS_3_ and VFLS_6_ groups

VFLS_3_ callus volume was 13% larger and VFLS_6_ callus volume was 55% larger than LS callus. VFLS_3_ callus density was 2% higher and VFLS_6_ callus density was 10% lower than that of LS callus. While there was a similar amount of callus at the trans-cortex, at the cis-cortex VFLS_3_ callus volume was 30% larger and VFLS_6_ was 112% larger compared to LS (Fig. 6, Table 1). At the gap level, the amount of callus at the cis cortex was significantly lower than that at the trans-cortex in LS samples while this difference was not significant in VFLS_3_ and VFLS_6_ samples (Fig. 7).

**Fig. 8:**
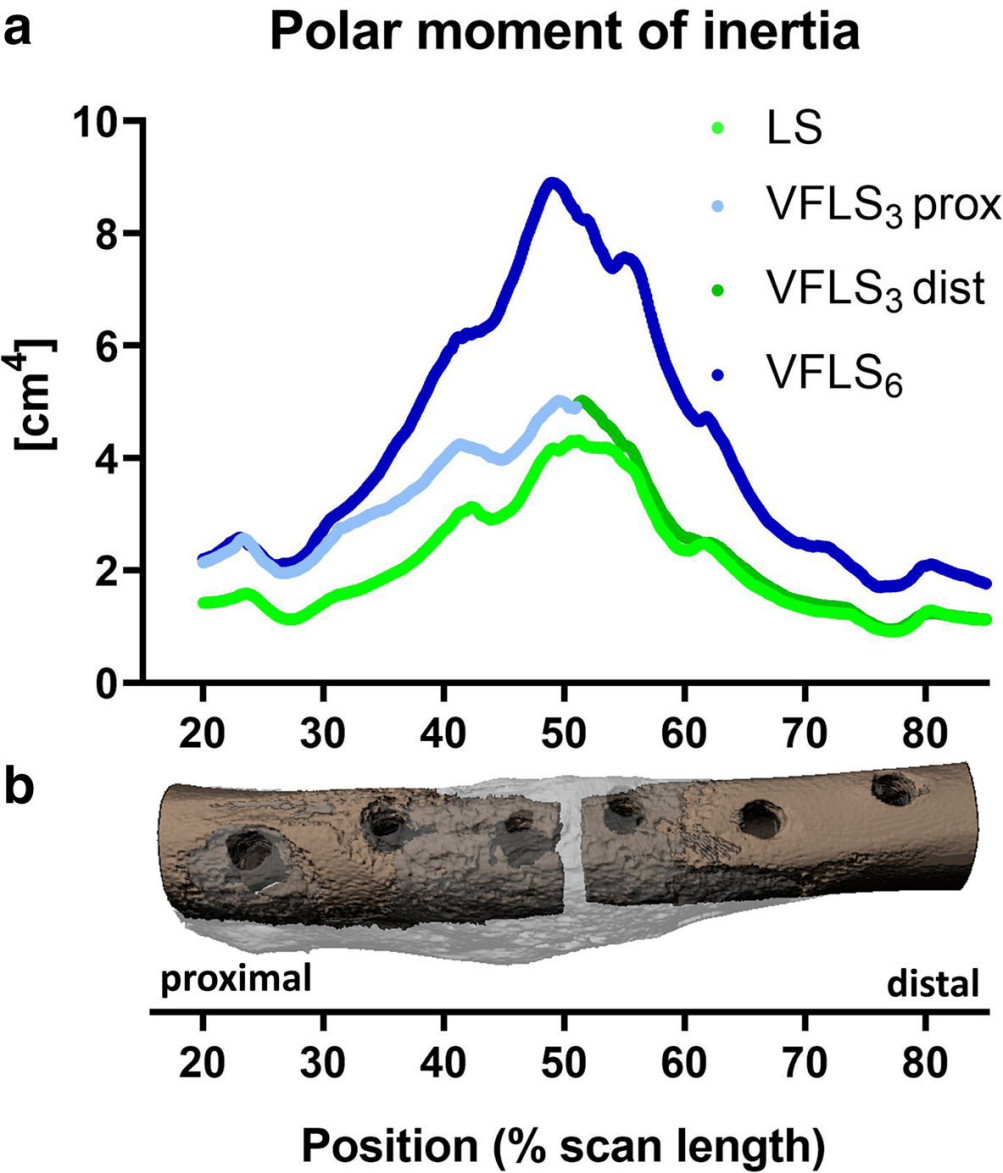
Panel **a**: the average cross-sectional polar moment of inertia (pMOI) measured along the bone axis in the three groups. On the x-axis the position of the investigated bone cross-sections with respect to the entire scan length. Panel **b**: a 3D reconstruction of a VFLS_3_ group sample. In this plot the fracture gap is centred around 50% of the scan. This plot shows that along the bone length, the callus generated by variable fixation is distributed farther from the bone axis (larger pMOI values). Furthermore, in bone segments implanted with VFLS the bone callus extends farther from the fracture gap. This is very evident in group VFLS_3_. For this group the pMOI values recorded in the proximal segment (VFLS_3_ prox) are larger than those in the proximal segment of the LS group. On the other side, in the distal segment (VFLS_3_ dist), where both groups featured standard locking screws, the pMOI profile is almost identical between VFLS_3_ and LS. The proximal to distal asymmetric bone callus formation can be appreciated on the VFLS_3_ samples depicted in panel B and confirms the observation made on standard X-rays

Plotting cross-sectional polar moments of inertia (pMOI) values according to their position along the bone length, showed a difference in callus spatial distribution between groups. Groups VFLS_3_ and VFLS_6_ showed larger values at the gap as well as in bone segments where VFLS have been implanted (Fig. 8).

#### Biomechanical testing

Overall, the biomechanical evaluation revealed similar values for all three groups. All implanted samples developed a crack passing through the fracture gap or at the interface between newly formed tissue and original bone but kept residual structural integrity. All contralateral samples completely failed with a multi-fragment spiral fracture. There was no significant difference in absolute and normalized values between groups for any of the investigated variables except for the normalized stiffness. Here, VFLS_6_ was significantly stiffer than VFLS_3_ (*p* = 0.036) (Table 2).

**Table 2:** Mean and standard deviation of absolute and normalized values for each investigated variable. There was no significant difference between groups for all investigated variable except for the normalized stiffness

**Absolute values**	**LS**	**VFLS**_**3**_	**VFLS**_**6**_	**Anova**	**VFLS**_**3**_/**LS**	**VFLS**_**6**_/ **LS**	**VFLS**_**6**_/**VFLS**_**3**_
Stiffness [Nm/degree]	4.9 ± 0.5	4.4 ± 0.4	5.4 ± 1.3	n.s.	−10.2%	10.2%	22.7%
Yield point [Nm]	36.2 ± 10.4	37.3 ± 6.9	38.4 ± 11.6	n.s.	3.0%	6.1%	2.9%
Ultimate strength [Nm]	40.1 ± 9.9	44.3 ± 10.0	42.3 ± 12.6	n.s.	10.5%	5.5%	−4.5%
Rot. to failure [degree]	10.2 ± 1.6	11.1 ± 2.3	10.0 ± 1.7	n.s.	8.8%	−2.0%	−9.9%
En. to failure [Nm*deg]	253.2 ± 85.2	285.7 ± 127.4	265.2 ± 93.1	n.s.	12.8%	4.7%	−7.2%
**Normalized values**	**LS**	**VFLS**_**3**_	**VFLS**_**6**_	**Anova**	**VFLS**_**3**_**/LS**	**VFLS**_**6**_**/LS**	**VFLS**_**6**_**/VFLS**_**3**_
Stiffness [%]	89.9 ± 8.4	74.2 ± 9.7	91.1 ± 13.0	*p* = 0.025	−17.5%	1.3%	22.8% (p = 0.036)
Yield point [%]	63.8 ± 25.9	71.7 ± 24.7	64.2 ± 27.8	n.s.	12.4%	0.6%	−10.5%
Ultimate strength [%]	54.9 ± 11.3	61.3 ± 13.7	58.2 ± 13.4	n.s.	11.7%	6.0%	−5.1%
Rot. to failure [%]	69.3 ± 7.9	79.5 ± 13.9	71.4 ± 8.7	n.s.	14.7%	3.0%	−10.2%
Energy to failure [%]	43.0 ± 10.8	54.7 ± 24.3	47.2 ± 16.2	n.s.	27.2%	9.8%	−13.7%

#### Histology

Minimal structural changes, characterized by slight activation of the germinative zones, and a minimal quantity of inflammatory cells were found in the popliteal and inguinal lymph nodes in both treated and non-treated limbs of groups VFLS_6_ and LS. In 11/12 animals of both groups, no intra or extracellular foreign material could be found in either of the lymph nodes. In one VFLS_6_ animal, unidentified brownish-beige small dotlike particles were observed in the cytoplasm of foamy (epithelioid-like) macrophage aggregates detected in the inguinal lymph node. This intracellular material was not polarizing under the microscope, and therefore could not be specified, neither as wear particles nor sleeve material. The Ziehl-Neelsen staining for acidophilic bacteria was negative.

At the level of the defects left from the screws, the analysis of the local tissue effects according ISO10993-6:2016 showed a minimal difference between tissue reaction at the trans-cortex between VFLS and LS. At the cis-cortex VFLS elicited a slight reaction compared standard locking screws indicating normal biodegradation and tissue remodeling. Overall, at both cortices and in the bone marrow cavity, no polymorphonuclear cells, no eosinophils, no necrosis and no osteolysis were observed in any sample. These same results were confirmed in group VFLS_3_ comparing the two types of screw in the same animals.

**Fig. 9:**
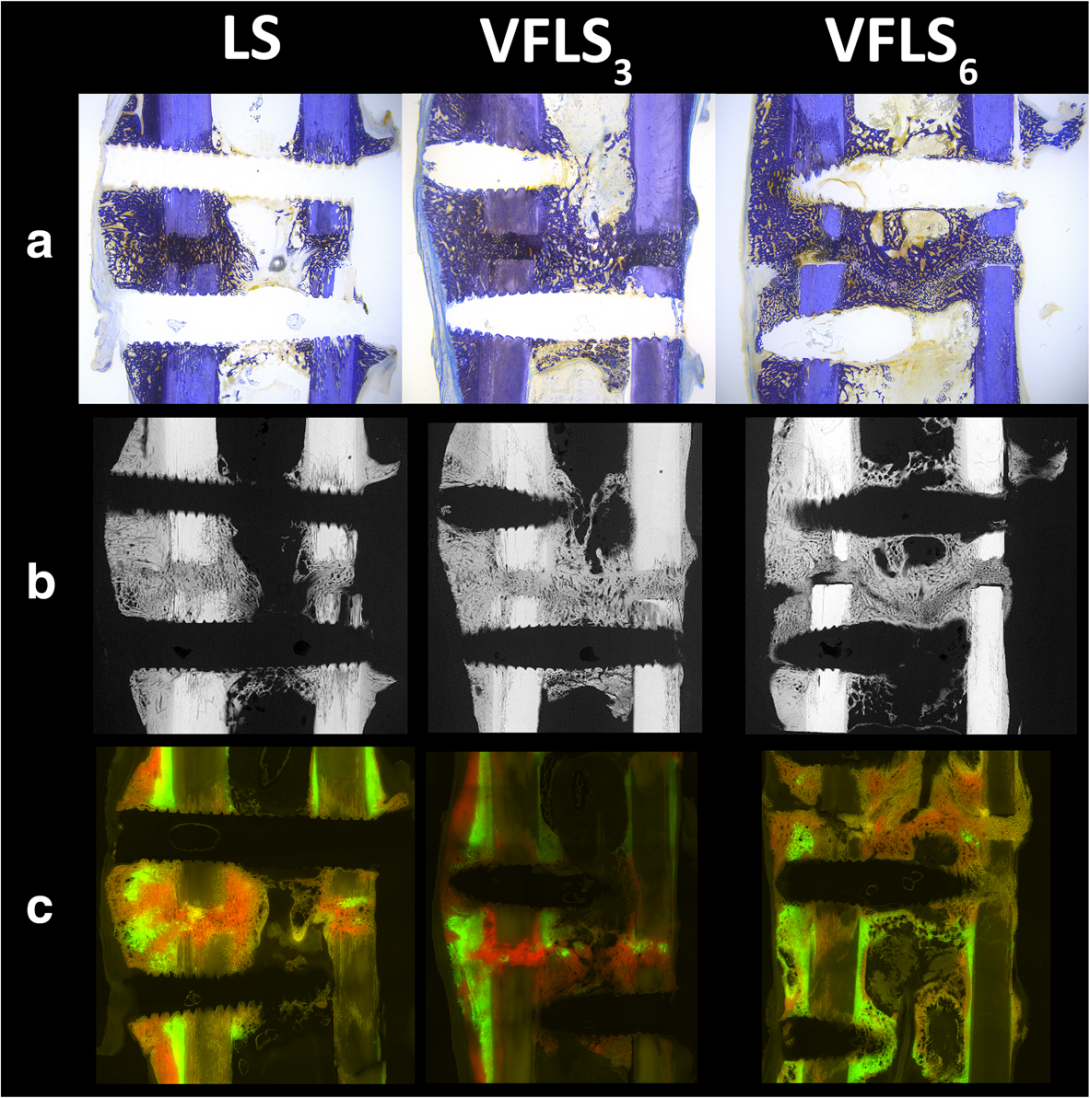
Panel **a**: Toluidine blue stained histology ground sections of one animal per group. The tissue around the test and reference implants has similar aspect both at the cis and trans-cortex. The fracture gap, still evident in all samples, is filled with disorganized calcified tissue. Overall, no significant differences were found between groups. Qualitatively we report more callus in the endosteal area in variable fixation groups. A lower percentage of old bone has been measured in variable fixation groups (VFLS_6_ – 26.86%; VFLS_3_ – 28.44%) compared to standard fixation (LS - 30.28%), together with slightly more new bone tissue in variable fixation groups (VFLS_3_ – 34.62%; VFLS_6_ – 34.02%) compared to standard fixation (LS - 30.61%). Panel **b**: Micro radiographs of the sample displayed above corroborating the observation and structure of the calcified tissue. The appearance of the cortices is very similar among groups and shows their activation is not causing a disruption of the cortical pattern. Panel **c**: Fluorescence overlay images the same samples. Calcein green showed an almost equal deposition of new tissue 3 weeks into treatment, with a higher deposition rate at the trans-cortex in all groups. Highest scores for the intramedullary area were given for VFLS_6_ followed by LS and VFLS_3_. At 6 weeks xylenol orange showed slightly more deposition intramedullary and at the cis-cortex in variable fixation groups and comparable deposition at the trans-cortex. Nine weeks post-surgery, VFLS_6_ showed similar deposition of oxytetracycline gold at the cortices and intramedullary, while the LS group featured more intramedullary deposition than at the cortices and VFLS_3_ featured more deposition at the cortices and less intramedullary

Histomorphometric measurements of ground sections revealed comparable and good healing of the gap area for all three groups. In total sections as well as at the cis-, trans-cortex and in the endosteal region no significant differences were found among groups for any investigated variable. However, groups VFLS_3_ and VFLS_6_ featured a slightly lower percentage of old and slightly higher percentage of new bone matrix in all locations. Finally, there was no significant difference in deposition of fluorescence dyes at 3, 6 and 9 weeks (Fig. 9). In summary the VFLS groups had a tendency for larger area of callus formation and a more symmetrical distribution of the callus around the defect site at 6 and 9 weeks, but a lower callus density when 6 VFLS were used (group VFLS_6_). The combination with 3 LS and 3 VFLS (group VFLS_3_) showed a denser callus and slightly focused callus at the defect gap.

## Discussion

Variable fixation promoted the formation of a substantially larger amount of bone callus compared to standard rigid fixation. The observed difference in callus formation and its distribution between cortices among groups revealed by the current study nicely translates in vivo and validates the previous biomechanical findings [16]. Sleeve resorption effectively dynamized the fracture gap, causing a progressive, substantial, and different increase in interfragmentary displacements at the cis and trans cortices. A progressive decrease in construct stiffness and a targeted increase in interfragmentary displacements have an effect on the production and spatial distribution of bone callus. Implanting variable fixation on one bone segment led on the whole bone to a 40% larger callus with similar mineral density. Its spatial distribution along the bone segment was different though. There was a larger amount of bone callus around bone segments implanted with variable fixation (Fig. 8). This finding is confirmed by the observation made on radiographs where trans cortical bone sections featuring VFLS were activated and locally contributed to the stabilization of the bone fragments producing additional bone callus. This suggests that VFLS screws have also a biological effect on the bone as organ, stimulating healthy cortical bone sections not directly adjacent to the osteotomy. A healthy activated bone section features all tissues, cells and signalling necessary to orchestrate the repair of a defect and can potentially support the activity at the fracture gap. There as well, according to our data on the gap, variable fixation induced larger and more homogeneously distributed callus between cortices compared to standard locking technology. With a + 30% larger cis callus and a non-significant difference between the amount of callus at the two cortices, variable fixation seems to have the potential to address the clinical concern of a modest callus formation at the cis-cortex using standard locking fixation [22–24].

We have detected the expected [16] difference when using variable fixation on one or on both segments. The ratio between the amount of variable fixation and the fracture gap size has a biological effect. Doubling the magnitude of variable fixation, namely the condition reproduced in group VFLS_6_, produced an even larger callus (93% on whole bone and 55% on the gap) and a boosting effect on the formation of callus in the cis-cortex (163% on the whole bone and 112% at the gap), even larger than that at the trans-cortex. However, this came at the cost of 7–10% lower mineral density compared to both other groups suggesting that, on a 3 mm gap, the strain provided was probably still in the “window of opportunity” but close to the upper boundary for optimal callus mineralization.

Histology revealed a comparable good healing of the defect area in all groups, with variable fixation groups featuring slightly more active remodeling indicated through a higher percentage of new bone formation. Fluorescent sections shown comparable deposition and maturation of bone callus during time among all groups. The biomechanical testing proved this boosting effect on callus formation is not coming at the cost of lower mechanical competence of the restored bone structure. They also confirm the decrease in torsional stiffness linked with variable fixation [16] is not disturbing the fracture-healing process.

The production of a larger, homogeneously distributed, amount of callus, capable of physiological maturation and mechanical stability, and the activation of the entire bone organ, suggest that variable fixation has the potential to increase the chances to bridge the fracture gap through stimulating callus formation. This might increase the overall success rate of osteosynthesis especially in more complicated cases where bone stimulation would be making the difference.

Variable fixation did not raise concerns at the implantation site. In fact, compared to standard locking technology the soft tissues adjacent to the implants and regional lymph nodes did not show macroscopic abnormalities. At 9 weeks, the sleeves were completely resorbed. The histological findings indicated absence of acute or chronic-active inflammation and no necrosis, osteolysis or abnormal tissue reaction. VFLS screws induced minimal reaction at the trans-cortex, while at the cis-cortex they induced a slight local effect compatible with the degradation of a small volume of resorbable material.

The main outcome of the investigation performed on X-rays is that variable fixation will require a learning curve in X-ray interpretation. Histology has shown that, using variable fixation, the cortical tissue pattern is not disrupted but as the radiological aspect of the bone cortices and the cortices/forming callus grays intensity ratio is slightly different due to the superimposition of abundant callus, the establishment of new standard assessment criteria will be necessary.

**Fig. 10:**
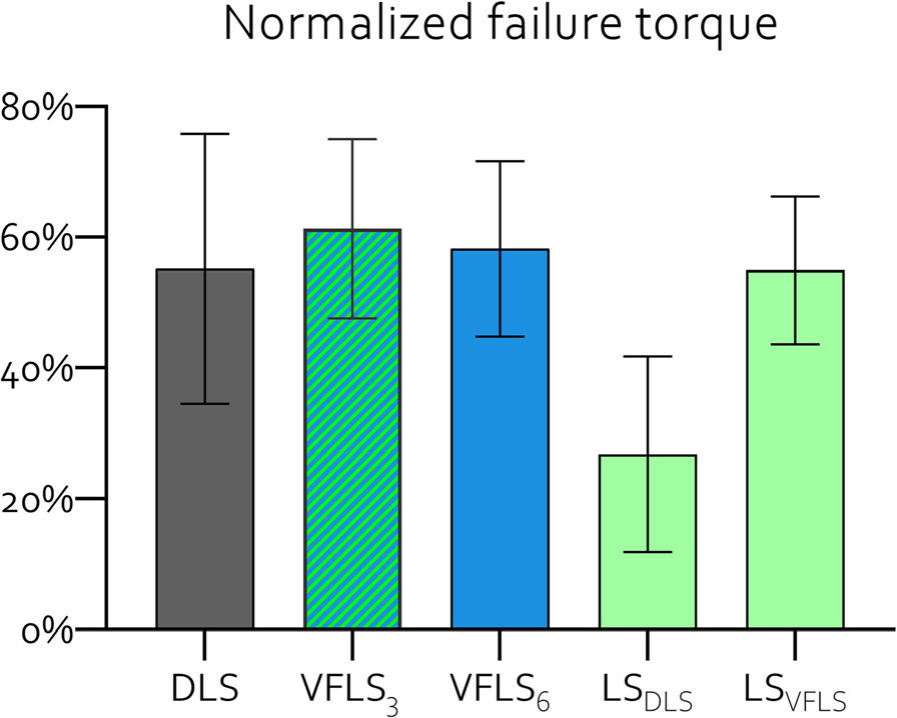
comparison between the normalized value collected in test and control groups tested with the same methods on this and a previous [21] publication

Comparison with similar investigations is challenging because of different endpoints [25, 26], usage of custom made prototypes [27, 28], lack of normalized data [27] or absolute data [21] and/or different methods for data analysis. In this study, no sample of the standard locking control group featured healing delays while in similar studies, 50% [27] or 80% [28] of standard locking control group featured deficient bridging with little or no new bone formation at the osteotomy gap and, in another study, 33% of samples featured very low failure torque [21]. Understanding why our normalized control group with standard locking screws outperformed published data of the same model [21] by about + 105% in failure torque (54.9% LS_VFLS_ versus 26.8% LS_DLS_) and about + 53% in torsional stiffness (89.9% LS_VFLS_ versus 58.89% LS_DLS_) is just speculation. However, despite variable fixation provided stronger bones compared to dynamic locking screws [21] the effect of control group data had clearly a major impact on the statistical conclusions of this study (Fig. 10).

To answer our research questions, we have investigated the influence of variable fixation on secondary fracture healing in a well-established sheep tibia transverse osteotomy model. Weekly radiographs, microCT, biomechanical testing and histology have been used to determine the amount, distribution and quality of bone callus at 9 weeks. We have used medical devices and not prototypes in order to provide surgeons with information they can directly use in their practice. We have tested combinations of technologies already fully characterized in a previous in vitro investigation to assess how their biomechanical behaviour translates in vivo. Using VFLS on one or two bone segments, we could strengthen the findings on local tissue reaction and validate the effect of two different magnitudes of variable fixation. Using widely used locking screws in the control group, we could compare the study outcome and conclusions with similar studies. However, this study has several limitations. The animal model used here did not reproduce soft tissues injuries typical of a traumatic event and it was not a delayed healing model. We have used a limited number of metabolically healthy animals. We did not monitor the interfragmentary movements during the 9 weeks but relied on published data to describe the mechanical behaviour of VFLS [16]. We also did not provide evidence the sleeve degrades progressively and in a fully controlled manner but relied here on the literature [17–20]. Biomechanical testing did not bring the samples until complete failure but stopped at the first 3 Nm drop in recorded resistant torque. Thus, we do not have a definitive information about the energy tolerated by the repaired bones until complete loss of their structural integrity. Histological data have been retrieved from the same samples previously tested for biomechanical properties and we did not present longitudinal data. This study was dedicated mainly to safety of using VFLS, which could be achieved with the current model.

## Conclusion

This is the first study testing variable fixation of an osteotomy in vivo. Our results show that variable fixation promoted the formation of a larger amount of mature bone callus, equally distributed between the cis and trans cortices. VFLS activated cortical sections of the bone as an organ, recruiting tissues, cells and signalling for contributing to the stabilization of the construct. The magnitude of variable fixation has a biological effect. Doubling the magnitude of variable fixation promoted the formation of an even larger bone callus. However, on the tested 3 mm gap this came with a slight decrease in mineral density suggesting that the usage of variable fixation needs always to be tuned with respect to the mass of the patient, the stiffness of the chosen bone plate and size of the fracture gap. At the implantation site Variable Fixation Locking Screws did not raise additional concerns with respect to standard locking screws. The degradation of the sleeve occurred within the 9 weeks study duration and did not elicit safety concerns on the bone cortex in contact and on the regional lymph nodes.

Variable fixation technology might be an additional tool for traumatology. The conditions where its usage can be most beneficial for patients needs to be clinically defined by surgeons. Longitudinal data and studies with clinical patients at risk of delayed fracture healing should be investigated in human clinical trials.

## Data Availability

The datasets used and/or analysed during the current study are available from the corresponding author on reasonable request.

## Abbreviations

LS: Group featuring six standard fixation locking screws/standard locking screw
VFLS: Variable Fixation Locking Screw
VFLS_3_: Group featuring Variable Fixation Locking Screws in the proximal fragment and standard fixation locking screws in the distal fragment
VFLS_6_: Group featuring six Variable Fixation Locking Screws
LS_VFLS_: Control group featuring six standard fixation locking screws tested in the study investigating VFLS
LS_DLS_: Control group featuring six standard fixation locking screws tested in the study investigating DLS
